# A Polymorphism rs12325489C>T in the LincRNA-ENST00000515084 Exon Was Found to Modulate Breast Cancer Risk via GWAS-Based Association Analyses

**DOI:** 10.1371/journal.pone.0098251

**Published:** 2014-05-30

**Authors:** Na Li, Ping Zhou, Jian Zheng, Jieqiong Deng, Hongchun Wu, Wei Li, Fang Li, Hongbin Li, Jiachun Lu, Yifeng Zhou, Chun Zhang

**Affiliations:** 1 Department of Genetics, Medical College of Soochow University, Suzhou, China; 2 Department of General Surgery, the Third Affiliated Hospital to Nantong University, Wuxi, China; 3 Department of Medical Oncology, The Third Affiliated Hospital of Harbin Medical University, Harbin, China; 4 The Institute for Chemical Carcinogenesis, The State Key Lab of Respiratory Disease, Guangzhou Medical University, Guangzhou, China; IFOM, Fondazione Istituto FIRC di Oncologia Molecolare, Italy

## Abstract

Breast cancer, one of the most common malignancies diagnosed among women worldwide, is a complex polygenic disease in the etiology of which genetic factors play an important role. Thus far, a subset of breast cancer genetic susceptibility loci has been addressed among Asian woman through genome-wide association studies (GWASs). In this study, we identified numerous long, intergenic, noncoding RNAs (lincRNAs) enriched in these breast cancer risk-related loci and identified 16 single nucleotide polymorphisms (SNPs) located within the sequences of lincRNA exonic regions. We examined whether these 16 SNPs are associated with breast cancer risk in 2539 cancer patients and 2818 control subjects from eastern, southern, and northern Chinese populations. We found that the C allele of the rs12325489C>T polymorphism in the exonic regions of lincRNA-ENST00000515084 was associated with a significantly increased risk of breast cancer (adjusted odds ratio [OR] = 1.79; 95% confidence interval [CI] = 1.50–2.12), compared with the rs12325489TT genotype. Biochemical analysis demonstrated that the C to T base change at rs12325489C>T disrupts the binding site for miRNA-370, thereby influencing the transcriptional activity of lincRNA-ENST00000515084 *in vitro* and *in vivo*, and affecting cell proliferation and tumor growth. Our findings indicate that the rs12325489C>T polymorphism in the lincRNA-ENST00000515084 exon may be a genetic modifier in the development of breast cancer.

## Introduction

As one of the most common malignant tumors in women throughout the world, the incidence and mortality of breast cancer has continued to rise in developing countries over the past decade [1], [2]. Epidemiological surveys have demonstrated that unfavorable environmental exposure, lifestyle habits, and genetic factors are closely related with the incidence of breast cancer [3]–[7]. However, only a small proportion of exposed individuals develop breast cancer, suggesting that genetic factors, such as genetic variants (GVs) plays a more important role in an individual’s risk of diseases [8], [9]. Much attention has been focused on the expression of protein-coding genes; however, accumulating evidence points to the essential role of non-protein-coding RNA (ncRNA) in cellular processes and aberrant ncRNA expression in disease phenotypes [10]–[12].

Transcriptome analysis has shown that the majority of human genome transcripts consist of ncRNA, including short non-coding RNAs (microRNAs), small interfering RNAs (siRNAs), piwi-interacting RNAs (piRNAs) and long ncRNAs (lncRNAs). However, to date, less is known about the role of the majority of noncoding transcripts, represented by lncRNAs, in development and in cancer biology. Long intergenic non-coding RNA (lincRNAs) are 100–200 nucleotides or longer transcript units that are interspersed between known protein-coding loci, but do not encode proteins [13], [14]. Recently, several studies have shown that groups of lincRNAs, correlated with various biological processes, including embryonic stem cell pluripotency [15], [16], gene transcription [17], [18] and chromatin-remodeling [19]. Furthermore, emerging evidence of dysregulated lincRNA expression in numerous cancers is increasing, suggesting a major role for involvement of lincRNA in human tumorigenesis and metastasis [20], [21]. A previously uncharacterized RNA gene, named *PTCSC3*, which acts as a tumor-suppressor, is involved in the predisposition to papillary thyroid carcinoma [10]. In addition, another lincRNA, *MALAT1*, was associated with a variety of human cancers of the breast, prostate and colon [22]–[25]. Yet, the molecular mechanism underlying the specific role of lincRNAs in cancer development has remained unclear.

Over the past few years, advances in genome-wide association studies (GWASs) have enabled the capture of thousands of genetic variants related to diseases and/or traits by high throughput technologies; however, at least one-third of the identified variants are within non-coding intervals [26]. Recently, several GWAS studies conducted among Asian women have reported several susceptibility risk loci that are associated with breast cancer risk [27]–[30]. Among these were numerous lincRNAs. Several relevant polymorphic sites located in the exonic regions of lincRNAs that may associate with breast cancer were identified; however, the association between genetic variations in lincRNAs exons and cancer susceptibility has rarely been reported.

In this paper, we hypothesized that genetic variations in the exonic region of lincRNAs may affect the functions of lincRNAs and thereby may contribute to breast cancer. To test this hypothesis, we conducted a hospital-based case-control study to investigate the association between genetic polymorphisms in the exonic regions of lincRNAs and susceptibility to breast cancer in a Chinese population.

## Materials and Methods

### Study Subjects

All subjects in the current study were ethnically homogenous Han Chinese women, and included 2539 breast cancer patients and 2818 healthy controls derived from eastern, southern, and northern Chinese populations. In the eastern Chinese population, patients (n = 1006) with diagnosed breast cancer were recruited consecutively from 2001 to 2009 from the First Affiliate Hospital of Soochow University (Suzhou, China), and were from Suzhou city and its surrounding regions, with a response proportion of 89%. There were no age, stage, or histology restrictions. To facilitate the study of breast cancer genetic susceptibility in a homogeneous population, 1018 cancer-free women were selected from a database consisting of 3,500 individuals based on a physical examination conducted in Jiangsu Province during the same time period that the cases were collected [31]. In the southern Chinese population, breast cancer cases (n = 772) were recruited from the Tumor Hospitals affiliated to Guangzhou Medical College between 2002 and 2009, with a response rate of 91%. Controls (n = 820) were selected from a pool of 5000 individuals who participated in a community-based screening program involving a health checkup conducted in Guangdong province during the same time period that the cases were recruited [32]. In the northern Chinese population, patients (n = 761) with diagnosed breast cancer were recruited from the Third Affiliate Hospital of Harbin Medical University between 2005 and 2010, and were from Harbin city and its surrounding regions. There were no age, stage, or histology restrictions. Similar to the southern Chinese population, controls (n = 980) were selected from a pool of 6000 individuals who had participated in a community-based screening program involving a health checkup [33]. Furthermore, the selection criteria for all controls were that they had no history of cancer, and were frequency matched to each set of cases on age (±5 years), with a response proportion of 90%. The tumor, node, metastasis (TNM) classification and tumor staging were evaluated according to the 2002 American Joint Committee on Cancer Staging system. At recruitment, after giving a written informed consent, each subject was scheduled for an interview with a structured questionnaire to provide data on age, body mass index, family history of cancer and other factors, and none had blood transfusion in the last 6 months to provide a blood sample for DNA analysis. The study was approved by the medical ethics committee of Soochow University, and the institutional review boards of Guangzhou Medical University and the Harbin Medical University. Clinical features of the patients are summarized in **Table 1**.

**Table 1:** Characteristics of breast cancer patients and controls in Chinese populations used for association study.

Characteristics	Suzhou population		Guangzhou population		Harbin population		Overall	
	Cases n (%)	Controls n (%)	Cases n (%)	Controls n (%)	Cases n (%)	Controls n (%)	Cases n (%)	Controls n (%)
**Age (years)**								
≤60	842 (83.7)	919 (90.3)	684 (88.6)	719 (87.7)	664 (87.3)	801 (81.8)	2190 (86.3)	2439 (86.6)
>60	164 (16.3)	99 (9.7)	88 (11.4)	101 (12.3)	97 (12.7)	179 (18.2)	349 (13.7)	379 (13.4)
**Age at menarche (years)**								
≤14	531 (52.8)	668 (65.6)	409 (53.0)	416 (50.7)	423 (55.6)	602 (61.4)	1363 (53.7)	1686 (59.8)
>14	475 (47.2)	350 (30.4)	363 (47.0)	404 (49.3)	338 (44.4)	378 (38.6)	1176 (46.3)	1132 (40.2)
**Body mass index**								
≤20	398 (39.5)	150 (14.7)	196 (25.4)	120 (14.6)	220 (28.9)	213 (21.8)	814 (32.1)	483 (17.1)
20–28	575 (57.2)	762 (74.9)	530 (68.6)	599 (73.1)	513 (67.4)	704 (71.8)	1618 (63.7)	2065 (73.3)
≥28	33 (3.3)	106 (10.4)	46 (6.0)	101 (12.3)	28 (3.7)	63 (6.4)	107 (4.2)	270 (9.6)
**Menstrual history**								
Premenopause	621 (67.1)	553 (54.3)	396 (51.3)	378 (46.1)	375 (49.3)	413 (42.1)	1392 (54.8)	1344 (47.7)
Menopause	385 (38.2)	465 (45.7)	376 (48.7)	442 (53.9)	386 (50.7)	567 (57.9)	1147 (45.2)	1474 (52.3)
**Family history of cancer**								
Positive	104 (10.3)	86 (8.4)	77 (10.0)	59 (7.2)	76 (10.0)	80 (8.2)	257 (10.1)	225 (8.0)
Negative	902 (89.7)	932 (91.6)	695 (90.0)	761 (92.8)	685 (90.0)	900 (91.8)	2282 (89.9)	2593 (92.0)
**Pathological type**								
Invasive ductal carcinoma	865 (86.0)		564 (73.1)		603 (79.2)		2032 (80.0)	
Other carcinoma	141 (14.0)		208 (26.9)		158 (20.8)		507 (20.0)	
**Stage**								
I	272 (27.0)		163 (21.1)		190 (25.0)		625 (24.6)	
II	624 (62.0)		436 (56.5)		457 (60.1)		1517 (59.7)	
III	93 (9.2)		101 (13.1)		89 (11.7)		283 (11.2)	
IV	17 (1.8)		72 (9.3)		25 (3.2)		114 (4.5)	
**Estrogen receptor status**								
Positive	547 (54.4)		445 (57.6)		433 (56.9)		1425 (56.1)	
Negative	459 (45.6)		327 (42.4)		328 (43.1)		1114 (43.9)	
**Progesterone receptor status**								
Positive	574 (57.1)		466 (60.4)		460 (60.4)		1500 (59.1)	
Negative	432 (42.9)		306 (39.6)		301 (39.6)		1039 (40.9)	
**HER-2 status**								
Positive	124 (12.3)		120 (15.6)		105 (13.8)		349 (13.7)	
Negative	695 (69.1)		538 (69.7)		498 (65.4)		1731 (68.2)	
Unindentified	187 (18.6)		114 (14.7)		158 (20.8)		459 (18.1)	

### SNP Selection

We searched for all published literature investigating an association between genetic susceptibility and breast cancer risk up to April 2013 using the PubMed database and Web of Science. Relevant search terms were “genome-wide association study”, “GWAS”, “breast cancer”, “breast carcinoma”, “breast neoplasms”, and “Asian”. We also searched the reference lists in selected articles. We firstly excluded some articles by scanning the titles and abstracts of studies. Then, after reading the full text of the remaining articles, we identified a final set of studies. The papers included in this set had to meet the following criteria: (1) the outcome investigated was based on GWAS in relation to breast cancer in humans; (2) the articles were published in English; (3) the latest studies were selected among overlapping data and duplicated data; (4) GWAS was conducted using chip technology. In the obtained 4 GWAS articles, only significant associations between genetic susceptibility loci and breast cancer risk were included. Finally, using this search strategy, 4 common genetic susceptibility loci (6q25.1, 16q12.1, 10q21.2, 11q24.3) that were independently associated with breast cancer risk among Asian women in GWASs were identified. The selected studies are shown in **Table S1**. Using bioinformatics analysis software programs (HapMap Data Release 27 Phase II+III, February 2009, on NCBI B36 assembly, dbSNP b126 and UCSC Genome Browser-hg18 assembly), we identified eight lincRNAs that did not overlap with any recognized genes. Two lincRNAs located at the genetic susceptibility loci 6q25.1(chr6∶149.100.001–152.600.000), four lincRNAs at the loci 16q12.1(chr16∶45.500.001–51.200.000), and two lincRNAs at the loci 11q24.3 (chr11∶127.400.001–130.300.000) from the human lincRNAs database from a leading work of Ulitsky et al. [34], in which the authors combined long (>200 bp) noncoding transcripts from Ensembl, RefSeq, UCSC genes and obtained sets of 2,458 human lincRNAs. None of lincRNAs located at the loci 10q21.2 (chr10∶61,200,001–64,800,000). Furthermore, Using the dbSNP database (http://www.ncbi.nlm.nih.gov/), bioinformatics analysis software program (Haploview software 4.2) and the criteria of minor allele frequencies of greater than 5% in the Chinese population, 16 SNPs located on exons of the selected 8 lincRNAs were finally extracted (6q25.1: rs2272901C>T, rs532367A>G, rs603092G>A; 16q12.1: rs17841343G>A, rs4785367T>C, rs2278016T>C, rs3815784C>T, rs1362378A>C, rs12325489C>T, rs3803662T>C; 11q24.3: rs10750417G>A, rs10894115C>G, rs1814343T>C, rs1814344T>C, rs4937447C>T, rs10894116G>A) (**Table 2**).

**Table 2:** Summary of results for selected SNPs showed associations with risk of breast cancer.

Loci	Chr:position^a^	LincRNA:position^a^	MAF (case, control)	OR (95%CI)^b^	*P* value
**Eastern Chinese (Suzhou)**					
rs12325489(C>T)	16q:50864577	ENST00000515084 exon 1	T = 0.29, 0.38	1.49 (1.31–1.68)	**<1.0×10^−7^**
rs2272901(C>T)	6q:149581466	uc003qmi.2 exon 2	T = 0.41, 0.41	1.09 (0.96–1.25)	0.182
rs532367(A>G)	6q:149584491	uc003qmi.2 exon 3	G = 0.45, 0.46	1.02 (0.90–1.16)	0.679
rs603092(G>A)	6q:149584784	uc003qmi.2 exon 3	A = 0.13, 0.14	1.12 (0.93–1.36)	0.469
rs17841343(G>A)	16q:48512623	ENST00000506071 exon 1	A = 0.21, 0.21	0.98 (0.84–1.15)	0.915
rs4785367(T>C)	16q:48513695	ENST00000506071 exon 1	C = 0.28, 0.30	1.11 (0.96–1.28)	0.071
rs2278016(T>C)	16q:50674510	uc002egu.2 exon 1	C = 0.13, 0.13	0.97 (0.80–1.18)	0.882
rs3815784(C>T)	16q:50675154	uc002egu.2 exon 1	T = 0.07, 0.06	0.78 (0.60–1.02)	0.066
rs1362378(A>C)	16q:50863415	ENST00000515084 exon 1	C = 0.45, 0.45	1.01 (0.89–1.15)	0.943
rs3803662(T>C)	16q:51143842	NR_033920 exon 1	C = 0.35, 0.35	0.96 (0.83–1.10)	0.913
rs10750417(G>A)	11q:128987385	ENST00000511090 exon 1	A = 0.21, 0.20	0.92 (0.79–1.09)	0.313
rs10894115(C>G)	11q:129070287	uc001qfd.1 exon 1	G = 0.23, 0.22	0.91 (0.78–1.06)	0.148
rs1814343(T>C)	11q:129070462	uc001qfd.1 exon 1	C = 0.39, 0.39	1.00 (0.88–1.15)	0.915
rs1814344(T>C)	11q:129070626	uc001qfd.1 exon 1	C = 0.24, 0.23	0.98 (0.84–1.14)	0.513
rs4937447(C>T)	11q:129070761	uc001qfd.1 exon 1	T = 0.47, 0.45	0.88 (0.77–1.01)	0.146
rs10894116(G>A)	11q:129072920	uc001qfd.1 exon 1	A = 0.16, 0.18	1.09 (0.91–1.29)	0.358
**Southern Chinese (Guangzhou)**					
rs12325489(C>T)	16q:50864577	ENST00000515084 exon 1	T = 0.29, 0.39	1.52 (1.31–1.79)	**<1.0×10^−7^**
**Northern Chinese (Harbin)**					
rs12325489(C>T)	16q:50864577	ENST00000515084 exon 1	T = 0.26, 0.36	1.58 (1.34–1.82)	**<1.0×10^−7^**
**Pooled analysis**					
rs12325489(C>T)	16q:50864577	ENST00000515084 exon 1	T = 0.28, 0.37	1.51 (1.37–1.68)	**<1.0×10^−7^**

a Data of position based on the International HapMap project (Rel 27 PhaseII+III, Feb09, on NCBI B36 assembly, dbSNP b126).

b Data were calculated by unconditional logistic regression, adjusted for age, BMI, and family history of cancer.

### Genotyping Analysis

Genome DNA was extracted from peripheral blood lymphocytes of the study subjects. Allele-specific MALDI-TOF mass spectrometry was used to genotype the markers used in the association analyses, as previously described [7]; genotyping of all markers was performed by MassArray (Sequenom, SanDiego, CA). All breast cancer patients and healthy controls in Suzhou center were genotyped for the 16 polymorphisms; patients and controls from Guangzhou and Harbin centers were genotyped only for the polymorphism rs12325489 to verify the results obtained in the Suzhou population. The genotyping results were further confirmed by direct sequencing.

### Animals and Cell Culture

Twenty female BALB/c nude mice that were 4–5 weeks of age and each weight (20±2)g were purchased from the Shanghai Laboratory Animal Center at the Chinese Academy of Sciences (Shanghai, China). The mice were allowed to acclimate to local conditions for at least 1 week and were maintained on a 12 h light-dark cycle with food and water *ad libitum*. This study was carried out in strict accordance with the recommendations in the Guide for the Care and Use of Laboratory Animals of the National Institutes of Health. The protocol was approved by the Committee on the Ethics of Animal Experiments of the Laboratory Animal Center of Soochow University (Permit Number: SYXK(SHU20120045)). Human breast cancer cell lines (MCF-7 and Bcap-37) were purchased from the Cell Bank of Type Culture Collection of the Chinese Academy of Sciences, Shanghai Institute of Cell Biology, and were passaged for fewer than 6 months. MCF-7 and Bcap-37 cells were cultured in RPMI 1640 medium (Gibco-BRL, Gaithersburg, MD, USA) supplemented with 10% fetal bovine serum (Gibco-BRL, Gaithersburg, MD, USA) and 1×antibiotics/antimycotics, at 37°C in the presence of 5% CO_2_.

### *In-silico* Prediction of Folding Structures Induced by rs12325489C>T in lincRNA-ENST00000515084

It is plausible that certain structures are more likely to play key roles in biological functions; thus, structural rearrangement may influence the expression and functions of genes by affecting its folding structures. We used RNAfold [35] and SNPfold algorithms [36], [37] to predict the putative influence of rs12325489C>T on the local folding structures of lincRNA-ENST00000515084 by analyzing the 61-bp regions flanking the polymorphism.

### Subcellular Fractionation

Cells from 2 different breast cancer cell lines, namely, Bcap-37 and MCF-7, were cultured in a humidified incubator for 2 days. For subcellular fractionation experiments, up to 2×10^6^ cells were used. Cytosolic and nuclear extracts from breast cancer cells were collected using a Nuclear/Cytosol Fractionation kit (Biovision, USA) according to the manufacturer’s instructions. Briefly, Bcap-37 and MCF-7 cells were lysed with a buffer containing 10 mM Tris-HCl (pH = 7.4), 100 mM NaCl, 2.5 mM MgCl2, and 40 mg/ml digitonin for 10 min. The resulting lysates centrifuged with 2,060×g for 10 min at 4°C. The supernatant was used for the cytosolic fraction. Subsequently, the pellets were washed and incubated with RIPA buffer at 4°C for 10 min. After centrifugation at 4°C for 10 min at 2,060×g, the nuclear fraction was collected.

### Construction of Reporter Plasmids

C-allelic reporter constructs were prepared by amplifying the lincRNA exonic region spanning the 258 bp flanking the rs12325489 polymorphism from subjects homozygous for the C allele (rs12325489CC) with the forward primer 5′-CCGCTCGAGCCATTGGTAAGAAGCA-3′ and the reverse primer 5′-ATTTGCGGCCGCCTTTGAATAGGGAAGAAC-3′, which included *Xho*I and *Not*I (Fermentas, Hanover, MD, USA) restriction enzyme sites, and cloning these fragments into psiCHECK-2 (Promega, Madison, WI, USA). A Quick Change XL site-directed mutagenesis kit (Stratagene, La Jolla, CA) was used to obtain rs12325489T reporter constructs from the psiCHECK-2-rs12325489C constructs by site-directed mutagenesis as described previously [38]. The amplified exonic regions including the rs12325489C>T polymorphism were inserted into the *Xho*I and *Not*I enzyme sites of the 3′-UTR of the *Renilla* luciferase gene in the vector psiCHECK-2. Finally, the resulting constructs (psiCHECK-2-rs12325489T and psiCHECK-2-rs12325489C) were sequenced to confirm the allele, orientation, and integrity of each insert.

### Transient Transfections and Luciferase Assays

Bcap-37 and MCF-7 cells were seeded in 24-well plates (1×10^5^ cells per well) and cultured to 60–70% confluence before transfection; cells were then transfected with the reporter plasmids described above using Lipofectamine 2000 (Invitrogen, CA, USA). In each well, co-transfection was performed using 800 ng of constructed plasmid DNA and 0, 1, or 40 pmol miRNA-370 mimics (Shanghai GenePharma Co., Ltd.), and with or without 40 pmol miRNA-370 inhibitor, according to the manufacturer’s instructions. Additionally, for every miRNA transfection experiment, 100 pmol of non-specific miRNA (GenePharma Co., Ltd.) was used as a negative control. After transfection for 24 h, 100 µL luciferase assay reagent was added to assay the cells. Luciferase activity was measured with the Dual-Luciferase Reporter assay system (Promega, Madison, WI, USA) using a TD-20/20 luminometer (Turner Biosystems, Sunnyvale, CA, USA) according to the manufacturer’s instructions, and the results were normalized against the activity of the *Renilla* luciferase gene. Each group included 6 replicates, and independent triplicate experiments were performed.

### Real-time PCR Analysis

Thirty-nine breast cancer tissue specimens were obtained from biopsies of patients and were stored in liquid nitrogen before analysis. Each subject signed a written consent approved by the medical ethics committee of Soochow University. Total RNA was obtained from these cancerous tissues with TRIzol reagent (Molecular Research Center, Inc). According to the manufacturer’s protocol, cDNA was generated from mRNA using the random primer and Superscript II (Invitrogen). Real-time quantitative polymerase chain reaction (PCR) was carried out to quantify the relative gene expression of lincRNA-ENST00000515084, using an ABI Prism 7500 sequence detection system (Applied Biosystems) based on the SYBR-green method. All quantifications were performed using *GAPDH* as an internal reference gene. The primers used for PCR amplification of the lincRNA-ENST00000515084 cDNA were 5′-CTCTTCCATCTGGCTGTTTCTAA-3′ (F) and 5′-GCTTCAAATGTTGATGGAAAGTC-3′ (R). For *GAPDH*, the primers used were 5′-GAAGGTGAAGGTCGGAGTC-3′ (F) and 5′-GAAGATGGTGATGGGATTTC-3′ (R). The lincRNA**-**ENST00000515084 expression measurements in individuals were normalized against the expression of *GAPDH,* using a modified method. In addition, we subsequently examined the relative level of lincRNA**-**ENST00000515084 in both the nucleus and cytoplasm of breast cancer cell lines. The All-in-One TM miRNA qRT-PCR Detection kit (GeneCopoeia, Rockville, MD, USA) was used to detect basal miRNA-370 expression *in* breast cancer tissues, according to the manufacturer’s protocol. The expression of miRNA-370 was calculated relative to the *U6* small nuclear RNA. All analyses were performed in a blinded fashion in which the laboratory personnel were unaware of the genotyping data.

### Actinomycin D Assay

Bcap-37 and MCF-7 cells were plated in 24-well culture plates (5×10^4^ cells per well). Sixteen hours after plating, the cells were transfected with 0, 1, or 40 pmol miRNA-370 mimics or with miRNA-370 inhibitor (Shanghai GenePharma Co., Ltd.). Twenty-four hours after transfection, cells were incubated with actinomycin D (Sigma) for 1, 2, or 3 h as previously described [39]. Actinomycin D was used at a final concentration of 2 µg/mL. Six replicates for each group and the experiment were repeated at least 3 times.

### Cell Viability Assay

In 96-well, flat-bottomed plates (BD Biosciences, Bedford, MA), 100 µL MCF-7 and Bcap-37 cells suspension (10,000 cells per mL) were aliquoted into each well. After transfection as described above for the actinomycin D assay and 1, 2 and 3 days of cultivation, cell viability was measured using cell viability was measured by Cell Counting Kit-8 (CCK-8) system (Dojindo Laboratory, Kumamoto, Japan) according to the manufacturer’s instructions. Briefly, 10 µl CCK-8 solution of each well was added, the plates were then incubated at 37°C for 1 h, and the absorbance of each well was read at 450 nm using a microplate reader (MRX; Dynex Technologies, West Sussex, United Kingdom). Six replicates for each group and the experiment were repeated at least 3 times.

### Lentiviral Production and Transduction

The precursor sequence of miRNA-370 was synthesized by the Genewiz Company (Suzhou, China) and then cloned into the lentiviral expression vector pLVX-IRES-neo (Clontech Laboratories Inc., San Francisco, CA, USA). The resulting construct (pLVX-IRES-neo-miRNA-370) was verified by direct sequencing. Using a 3-plasmid transient co-transfection method (Lenti-T HT Packaging Mix, Clontech), replication-defective VSV-G pseudotyped viral particles were packaged in human embryonic kidney cells LentiX 293T (Clontech Laboratories Inc.). The virus was then harvested and filtered. For transduction, MCF-7 and Bcap-37 cells were infected with the control lentivirus (empty vector containing only lentivirus without the miRNA-370 fragment) or miRNA-370 lentivirus. After 48 h of transduction, the cells were stably selected with G418 (500 µg/mL), and the drug-resistant cell populations were used for subsequent studies.

### Xenografts in Mice

Cells harboring the MCF-7-empty vector, MCF-7-miRNA-370, Bcap-37-empty vector, or Bcap-37-miRNA-370 were diluted to a concentration of 5×10^6^ cells per mL in physiological saline, and 0.1 mL of the suspension was injected subcutaneously into the posterior flank of mice. Five nude mice were used for each group. When a tumor was palpable, tumor growth was measured every other day following subcutaneous injection of tumor cells on one or both sides of the back of syngeneic mice, by measuring the 2 largest perpendicular diameters with calipers, and tumor volume was calculated according to the following formula: V = L×W^2^×0.5 (L, length; W, width).

### Statistical Analysis

The differences in the distributions of age, menstrual history, BMI, and family history of cancer between cases and cancer-free controls, as well as the allele and genotype frequencies were assessed by two-sided chi-squared tests. Unconditional logistic regression models was used to assess the association between the risk of breast cancer and each SNP by odds ratios (ORs) and their 95% confidence intervals (95%CIs), with adjustments for age, BMI, and family history. Logistic regression modeling was used in the trend test, as well as to evaluate the potential multiplicative and additive gene-gene and gene-environmental factor interactions. Furthermore, the data were further stratified by age, age at menarche (years), menstrual history, BMI, and family history of cancer to evaluate the stratum variable-related ORs among various lincRNA-ENST00000515084 rs12325489C>T genotypes, which were further tested for homogeneity within the strata. Statistical power was computed by applying the PS software (http://biostat.mc.vanderbilt.edu/twiki/bin/view/Main/PowerSampleSize, accessed Dec 14, 2010). Student’s t-test was used to compare the differences in the levels of luciferase reporter gene expression. Kruskal-Wallis one-way ANOVA test and linear regression models were used to evaluate the effect of various SNPs on the lincRNA-ENST00000515084 transcript expression. All tests were two-sided by using the SAS software (version 9.1; SAS Institute, Cary, NC, USA). A *P*<0.05 was used as the criterion for statistical significance.

## Results

### Genotypes and Risk of Breast Cancer

In the discovery set analysis of these sixteen SNPs genotyped in the eastern Chinese population, a significant association with breast cancer risk was observed for rs12325489C>T but not for the other (**Table 2**). Specifically, the adjusted ORs associated with the lincRNA-ENST00000515084 rs12325489CC and CT genotype were 2.08 (95%CI = 1.53–2.82) and 1.32 (95%CI = 0.97–1.80), when compared with the rs12325489TT genotype. These associations were confirmed in the southern and northern Chinese population, where the adjusted ORs of carrying the rs12325489CC and CT genotype were 2.20 (95%CI = 1.59–3.06) and 1.46 (95%CI = 1.05–2.03) in the southern Chinese population and 2.52 (95%CI = 1.84–3.71) and 1.67 (95%CI = 1.17–2.38) in the northern Chinese population, respectively, when compared with the rs12325489TT genotype (**Table 3**).

**Table 3:** Rs12325489C>T genotype among patients and controls and their association with risk of breast cancer.

Genotypes	Cases	Controls	OR (95%CI)^a^	*P* value
	n (%)	n (%)		
**Eastern Chinese (Suzhou)**	**n = 1006**	**n = 1018**		
rs12325489C>T				
TT	92 (9.1)	146 (14.3)	1.00 (reference)	<1.0×10**^−^** ^5^
CT	398 (39.6)	474 (46.6)	1.32 (0.97–1.80)	
CC	516 (51.3)	398 (39.1)	2.08 (1.53–2.82)	
CT+CC	914 (90.9)	872 (85.7)	1.67 (1.25–2.23)	
**Southern Chinese (Guangzhou)**	**n = 772**	**n = 820**		
rs12325489C>T				
TT	73 (9.5)	133 (16.2)	1.00 (reference)	<1.0×10**^−^** ^5^
CT	308 (39.9)	372 (45.4)	1.46 (1.05–2.03)	
CC	391 (50.6)	315 (38.4)	2.20 (1.59–3.06)	
CT+CC	699 (90.5)	687 (83.8)	1.80 (1.32–2.45)	
**Northern Chinese (Harbin)**	**n = 761**	**n = 980**		
rs12325489C>T				
TT	53 (7.0)	133 (13.6)	1.00 (reference)	<1.0×10**^−^** ^5^
CT	290 (38.1)	438 (44.7)	1.67 (1.17–2.38)	
CC	418 (54.9)	409 (41.7)	2.52 (1.84–3.71)	
CT+CC	708 (93.0)	847 (86.4)	2.12 (1.52–2.97)	
**Pooled analysis**	**n = 2539**	**n = 2818**		
rs12325489C>T				
TT	218 (8.6)	412 (14.6)	1.00 (reference)	<1.0×10**^−^** ^7^
CT	996 (39.2)	1284 (45.6)	1.43 (1.18–1.72)	
CC	1325 (52.2)	1122 (39.8)	2.20 (1.83–2.66)	
CT+CC	2321 (91.4)	2406 (85.4)	1.79 (1.50–2.12)	

a Data were calculated by logistic regression analysis with adjusted for age, BMI, and family history of cancer.

A stratified analysis assessing the associations between the lincRNA-ENST00000515084 rs12325489C>T genotypes and the risk of breast cancer was conducted. However, there was no significant association in any of the subgroups (**Table 4**).

**Table 4:** Stratification analysis of rs12325489C>T genotypes by selected variables in breast cancer patients and controls.

	Patients (n = 2539)				Controls (n = 2818)				Adjusted OR(95%CI)^a^ * CC+CT vs. TT	*P* value^b^ *
	TT n (%)		CC+CT n (%)		TT n (%)		CC+CT n (%)			
**Age (years)**										
≤47	104	(4.1)	1105	(43.5)	157	(5.6)	1006	(35.7)	1.66 (1.28–2.16)	0.38
>47	114	(4.5)	1216	(47.9)	255	(9.0)	1400	(49.7)	1.94 (1.54–2.45)	
**Age at menarche (years)**										
≤14	96	(3.8)	1267	(49.9)	201	(7.1)	1485	(52.7)	1.79 (1.39–2.31)	0.57
>14	122	(4.8)	1054	(41.5)	211	(7.5)	921	(32.7)	1.98 (1.56–2.52)	
**Menstrual history**										
Premenopause	123	(4.8)	1269	(50.0)	180	(6.4)	1164	(41.3)	1.60 (1.25–2.03)	0.15
Menopause	95	(3.8)	1052	(41.4)	232	(8.2)	1242	(44.1)	2.07 (1.61–2.66)	
**Body mass index (BMI)**										
≤20	67	(2.6)	747	(29.4)	74	(2.6)	409	(14.5)	2.02 (1.42–2.87)	0.64
20–28	140	(5.5)	1478	(58.2)	301	(10.7)	1764	(62.6)	1.80 (1.46–2.23)	
≥28	11	(0.4)	96	(3.8)	37	(1.3)	233	(8.3)	1.39 (0.63–2.83)	
**Family history of cancer**										
Positive	21	(0.8)	236	(9.3)	29	(1.0)	196	(7.0)	1.66 (0.92–3.01)	0.76
Negative	197	(7.8)	2085	(82.1)	383	(13.6)	2210	(78.4)	1.83 (1.53–2.20)	
**Pathological type**										
Invasive ductal carcinoma	170	(6.7)	1862	(73.3)	412	(14.6)	2406	(85.4)	1.88 (1.55–2.26)	0.47
Other carcinoma	48	(1.9)	459	(18.1)	412	(14.6)	2406	(85.4)	1.64 (1.19–2.24)	
**Stage**										
I/II	177	(7.0)	1965	(77.4)	412	(14.6)	2406	(85.4)	1.90 (1.58–2.29)	0.21
III/IV	41	(1.6)	356	(14.0)	412	(14.6)	2406	(85.4)	1.49 (1.06–2.09)	
**Estrogen receptor status**										
Positive	124	(4.9)	1301	(51.2)	412	(14.6)	2406	(85.4)	1.80 (1.45–2.22)	0.84
Negative	94	(3.7)	1020	(40.2)	412	(14.6)	2406	(85.4)	1.86 (1.47–2.35)	
**Progesterone receptor status**										
Positive	131	(5.2)	1369	(53.9)	412	(14.6)	2406	(85.4)	1.79 (1.45–2.20)	0.78
Negative	87	(3.4)	952	(37.5)	412	(14.6)	2406	(85.4)	1.87 (1.47–2.39)	
**HER-2 status**										
Positive	42	(1.7)	307	(12.1)	412	(14.6)	2406	(85.4)	1.25 (0.89–1.76)	0.07
Negative	138	(5.4)	1593	(62.7)	412	(14.6)	2406	(85.4)	1.98 (1.61–2.42)	
Unindentified	38	(1.5)	421	(16.6)	412	(14.6)	2406	(85.4)	1.90 (1.34–2.69)	

*Data were for the combined discovery and validation sets;

a ORs were adjusted for age, BMI and family history of cancer in a logistic regression model;

b *P* value of the test for homogeneity between stratum-related ORs for lincRNA-ENST00000515084 (rs12325489 CC+CT vs. TT genotypes).

### Cellular Characterization of lincRNA-ENST00000515084

**Figure 1:**
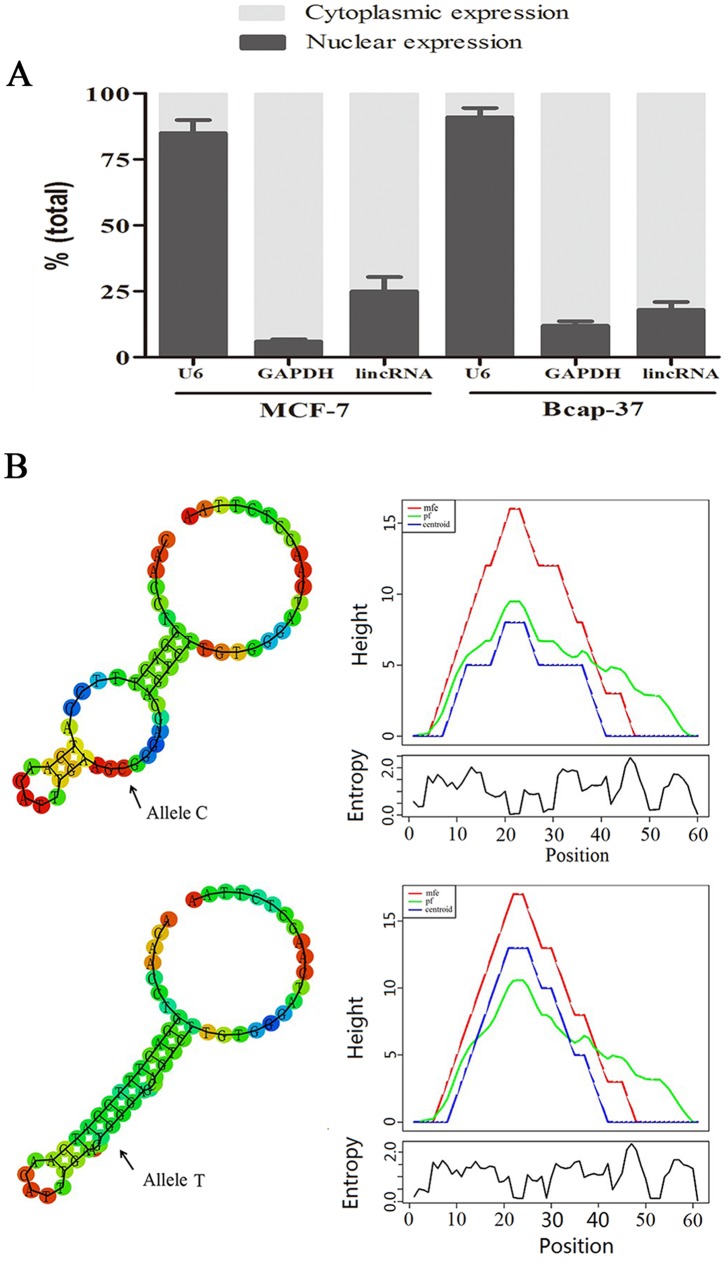
Cellular and molecular characterization of lincRNA-ENST00000515084. (**A**) The levels of nuclear control transcript (*U6*), cytoplasmic control transcript (*GAPDH* mRNA), and lincRNA-ENST00000515084 were assessed by RT-qPCR in nuclear and cytoplasmic fractions. Data are mean±standard error of the mean. Data are presented as a percentage of *U6*, *GAPDH* and lincRNA-ENST00000515084 levels and total levels for each were taken to be 100%. (**B**) *In-silico* prediction of folding structures induced by rs12325489C>T in lincRNA-ENST00000515084. The mountain plot is an xy-graph that represents a secondary structure including MFE structure, the thermodynamic ensemble of RNA structures (pf), and the centroid structure in a plot of height versus position. “mfe” represents minimum free energy structure; “pf” indicates partition function; “centroid” represents the centroid structure.

The levels of nuclear control transcript (*U6*), cytoplasmic control transcript (*GAPDH* mRNA), and lincRNA-ENST00000515084 were assessed by RT-qPCR in nuclear and cytoplasmic fractions, respectively. The results showed that *GAPDH* mRNA was exclusively detected in the cytoplasmic fraction, while nucleus-retained *U6* was predominantly found in the nuclear fraction. Further RT-qPCR analysis revealed that lincRNA-ENST00000515084 expression was predominantly cytoplasmic **(** **Figure 1A** **)**.

### *In-silico* Analysis of the Effect of rs12325489C>T on lincRNA-ENST00000515084 Folding

Using RNAfold and SNPfold algorithms in *in-silico* analysis, we predicted local structural changes of lincRNA-ENST00000515084 caused by the rs12325489C>T polymorphism located within the exonic region of lincRNA-ENST00000515084. As shown in **Figure 1B**, the results suggested that the C to T base change of rs12325489C>T affects the folding of lincRNA-ENST00000515084, which may affect the binding site for the microRNAs. This may then influence the transcriptional activity of the lincRNA*-*ENST00000515084 gene.

### Rs12325489C>T Genotypes Affect lincRNA-ENST00000515084 Expression by Inhibiting the Binding of miRNA-370 *in vitro*

**Figure 2:**
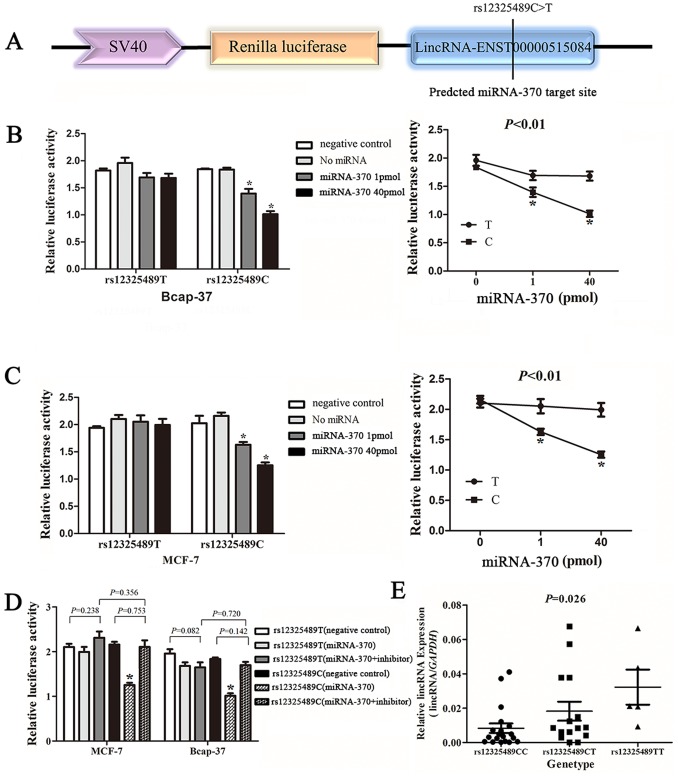
LincRNA-ENST00000515084 rs12325489C>T genotypes affect lincRNA-ENST00000515084 expression by regulating the miRNA-370 binding in *vitro.* (**A**) Reporter plasmids construction.Representative graph of luciferase activity of variant allele on luciferase reporter genes bearing the lincRNA-ENST00000515084 exonic region spanning 258 bp flanking the rs12325489C>T polymorphism segments in Bcap-37 (**B**) and MCF-7 cells (**C**). Relative luciferase activity of the psiCHECK-2-rs12325489T and psiCHECK-2-rs12325489C constructs co-transfected with miRNA-370 and inhibitor in Bcap-37 and MCF-7 cells (**D**). Renilla luciferase activity was measured and normalized to firefly luciferase. Six replicates were carried out for each group, and the experiment was repeated at least three times. Data are mean±standard error of the mean. Asterisk indicates a significant change (*P*<0.01). (**E**) LincRNA-ENST00000515084 expression level in thirty-nine breast cancer tissues for different rs12325489C>T genotypes (19 rs12325489CC, 15 rs12325489CT and 5 rs12325489TT); data are mean±standard error of the mean. lincRNA-ENST00000515084 expression normalized to *GAPDH* (*P* = 0.026 for lincRNA-ENST00000515084).

We used bioinformatics analysis programs (http://snpinfo.niehs.nih.gov/snpinfo/snpfunc.htm) to predict a binding site for human microRNA within the lincRNA-ENST00000515084 region that contains the rs12325489C>T polymorphism (**Figure 2A**). And the result showed that only miRNA-370 binds to lincRNA-ENST00000515084 transcripts containing the rs12325489C allele, while there are 4 microRNAs (miRNA-1229, miRNA-1260b, miRNA-617, miRNA-1260) that bind to lincRNA-ENST00000515084 transcripts containing the rs12325489T allele. The sequence of the predicted 5 microRNAs binding sites were presented in **Table S2**. These 5 microRNAs and either of the polymorphic reporters constructs were transiently co-transfected into Bcap-37 cells repectively, and luciferase activity subsequently assessed. The cells transiently co-transfected miRNA-370 mimics and construct containing the rs12325489C allele exhibited significantly reduced luciferase activity, in a concentration-dependent manner, compared with the construct containing the rs12325489T allele (**Figure 2B**). The same result was also observed when these experiments were repeated using MCF-7 cells (**Figure 2C**), but the other 4 microRNAs did not reveal significantly luciferase activity (**Figure S1**). The reporter vectors (psiCHECK-2-rs12325489C and psiCHECK-2-rs12325489T), miRNA-370 mimics, and miRNA-370 inhibitor were transiently co-transfected into Bcap-37 and MCF-7 cells. In both cell lines, no significant differences in luciferase activity were observed in the presence of the miRNA-370 inhibitor (**Figure 2D**). These results suggest that miRNA-370 can bind and negatively regulate the transcription of lincRNA-ENST00000515084 in the presence of rs12325489C allele.

### Association of lincRNA-ENST00000515084 rs12325489C>T Genotypes with lincRNA-ENST00000515084 Expression

We performed RT-qPCR to further evaluate the effects of rs12325489C>T on lincRNA-ENST00000515084 expression using 39 breast cancer tumor tissues with different genotypes. As shown in **Figure 2E**, patients with the rs12325489TT genotype expressed significantly higher lincRNA-ENST00000515084 mRNA levels (mean±SEM: 0.032±0.010), compared to carriers of the rs12325489CT (0.018±0.006) and rs12325489CC genotypes (0.008±0.003; ANOVA test: *P* = 0.026). And miRNA-370 is constitutively expressed in the 39 breast cancer tumor tissues; however, there was no significant association between the background expression of miRNA-370 and rs12325489C>T genotypes (0.026±0.003 for CC; 0.021±0.007 for CT and 0.026±0.008 for TT; ANOVA test: *P* = 0.810; linear regression test: *P* = 0.137) (**Figure S2**).

### Effects of the lincRNA-ENST00000515084 rs12325489C>T Genotypes on Cell Proliferation

**Figure 3:**
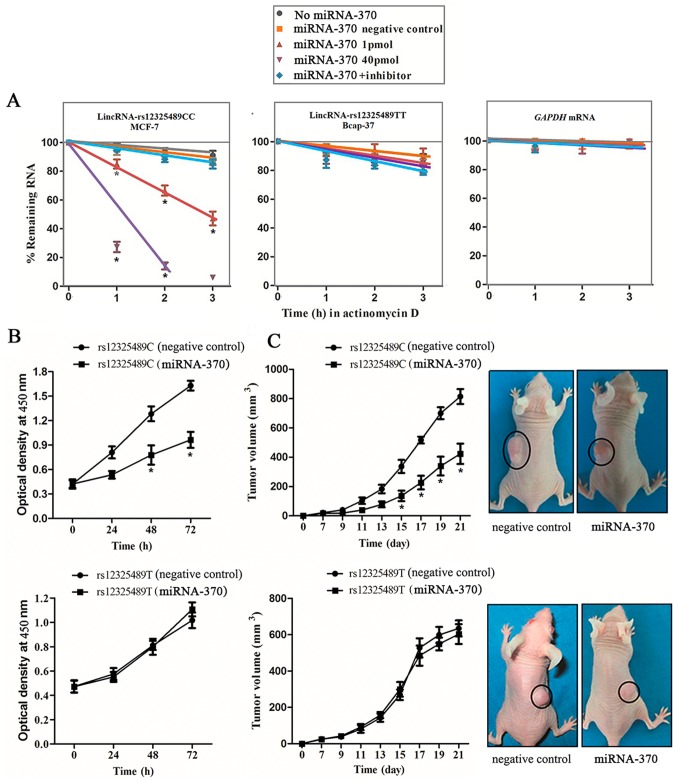
Effects of rs12325489C>T genotypes on cells’ proliferation and tumor growth. (**A**) The different steady-state of lincRNA-ENST00000515084 in rs12325489TT (Bcap-37) cells and rs12325489CC (MCF-7) cells. Twenty-four hours after transfecting with miRNA-370 mimics or miRNA-370 inhibitor, rs12325489TT (Bcap-37) cells and rs12325489CC (MCF-7) cells were incubated with actinomycin D for 1, 2 or 3 hours. The decline transcript levels of lincRNA-ENST00000515084 and *GAPDH* mRNA levels after actinomycin D treatment was measured by real-time PCR analysis; data are mean±standard error of the mean, normalized to *GAPDH*. *P<0.05 compared with control. (**B**) Cells’ proliferation rate was significantly inhibited in rs12325489CC (MCF-7) cells by binding miRNA-370. Cell proliferation was performed by the cell viability assay. Six replicates for each group and the experiment repeated at least three times. Data are mean±standard error of the mean (*P* = 0.001). *P<0.05 compared with control. (**C**) Effects of rs12325489C>T genotypes on tumor growth by binding miRNA-370. Figure shown is a representative of five nude mice for each group at the third week after xenograft-transplanted nude mouse tumor models of human breast cancer growth established. *P<0.05 compared with control.

DNA sequencing showed that MCF-7 and Bcap-37 cells harbor rs12325489CC and rs12325489TT genotypes, respectively. We further investigated the effect of lincRNA-ENST00000515084 rs12325489C>T genotypes on cell proliferation in *vitro*. Twenty-four hours after transfecting MCF-7 and Bcap-37 cells with miRNA-370 mimics or with miRNA-370 inhibitor, as shown in **Figure 3A**, the transcript levels of the lincRNA-ENST00000515084 rs12325489CC genotype (MCF-7 cells) was down-regulated from 100% to 85%±3.2% after RNA synthesis was blocked with actinomycin D for 1 h in the presence of 1 pmol miRNA-370. Furthermore, the remaining level of lincRNA-ENST00000515084 after exposure of cells to actinomycin D for 1 h in the presence of 40 pmol miRNA-370 was only 27%±3.6%. However, these treatments resulted in no significant change in transcript levels of lincRNA-ENST00000515084 with the rs12325489TT genotype in Bcap-37 cells (*P* = 0.623). In addition, cell viability assays revealed that cell proliferation rate was significantly inhibited in cells carrying the rs12325489CC genotype that had been transfected with miRNA-370 (*P* = 0.001). However, there were no noteworthy changes in cell growth rate in cells with the rs12325489TT genotype (*P* = 0.739) (**Figure 3B**).

### Effects of rs12325489C>T Genotypes on Tumor Growth by Binding to miRNA-370

We evaluated the effects of rs12325489C>T genotypes on tumor growth in xenografts model. Mice were injected with cells harboring the rs12325489CC (MCF-7)-empty vector, rs12325489CC (MCF-7)-miRNA-370, rs12325489TT (Bcap-37)-empty vector, or rs12325489TT (Bcap-37)-miRNA-370. As shown in **Figure 3C**, tumor growth from rs12325489CC (MCF-7)-miRNA-370 cell xenografts was delayed by 5 days compared to that of tumors formed from rs12325489CC (MCF-7)-empty vector cell xenografts, and the mean tumor volume after 3 weeks in the former group was about 424±69 mm^3^, approximately 391 mm^3^ smaller than those resulting from rs12325489CC (MCF-7)-empty cell xenografts. Growth of tumors from rs12325489TT (Bcap-37)-miRNA-370 cell xenografts was not inhibited when compared with that of tumors formed from rs12325489CC cells.

We further used 2 additional models, using rs12325489CC (MCF-7) and rs12325489TT (Bcap-37) wild-type cells, to investigate the general validity of our findings. The result showed that tumor growth was comparable to that of tumors formed from rs12325489CC (MCF-7)-empty vector and rs12325489TT (Bcap-37)-empty vector cell xenografts.

## Discussion

In the hospital-based case-control study containing 2539 breast cancer patients and 2818 controls, we found that the risk of breast cancer was significantly associated with the rs12325489C allele, which itself changes miRNA-370-mediated lincRNA-ENST00000515084 expression. Additionally, compared to the lincRNA-ENST00000515084 rs12325489T allele, the rs12325489C allele was correlated with an increase in the proliferation rate of breast cancer cells *in vitro* and *in vivo*.The association between rs12325489C>T polymorphism and breast cancer indicated the possibility that this genetic variant in lincRNA-ENST00000515084 exon may be a common susceptibility factor for developing breast cancer.

As one type of long non-coding RNA genes, lincRNAs are often tissue-specific mRNA-like transcripts lacking significant open reading frames [40], [41]. In various human tissues, lincRNAs are emerging as new players, associated with development in a stage-specific manner and diseases including cancers. It is biologically reasonable that functional lincRNAs may play a role in the development of cancer. One famous example is the lincRNA GAS5; genetic aberrations at this lincRNA locus have been found in many types of tumors, including melanoma, breast, and prostate cancers [42]–[44]. These lines of evidence suggest a crucial role of lincRNA in tumorigenesis. Recently, a subset of breast cancer susceptibility loci (6q25.1, 16q12.1, 10q21.2, and 11q24.3) has been identified among Asian women through GWASs. Bioinformatics analysis revealed several lincRNAs mapped to these genetic susceptibility loci. Our results are consistent with previous findings [45], [46], suggesting that dysregulation of lincRNA is associated with susceptibility to breast cancer in Chinese populations. Furthermore, cell proliferation experiments indicated that a subtle change in lincRNA-ENST00000515084 function due to the rs12325489C>T polymorphism may interfere with cell proliferation.

Cumulative evidence has demonstrated that numerous sets of lincRNAs themselves have distinct and critical biological functions in X-chromosome inactivation (*Xist, Tsix*) [47], [48], imprinting (*H19, Air*) [49], [50], regulation of gene expression (*HOTAIR*) [51], and reprogramming of human induced pluripotent stem cells [52], as indicated by gene expression patterns. A functional genomics approach and cell-based assays have demonstrated that specific lincRNAs could be transcriptionally regulated by key transcription factors in diverse biological processes [13]. In addition, recent studies have shown that some lincRNAs act as the precursor to microRNAs and are capable of regulatory function in response to cellular stress or oncogenic signals [53]. It is well recognized that non-coding RNAs play a regulatory role in several complex processes in the nucleus and cytoplasm [39], [54]. This difference in localization of lincRNAs suggests diverse mechanisms of regulation and function of lincRNAs involved in the nucleus and cytoplasm. Approximately 30% of embryonic stem cell lincRNAs are implicated in regulation in the nucleus and can be associated with multiple regulatory complexes to affect neighboring regions [55]. Alternatively, lncRNAs can participate in RNA-RNA interactions to carry out their regulatory roles in the cytoplasm [56]. Recently, it has been shown that the presence of a binding site for microRNA in the conserved site of a lincRNA gene may regulate lincRNA expression levels [34], [57], [58]. Our study showed that lincRNA-ENST00000515084 was moderately more abundant in the cytoplasm than in the nucleus of fractionated breast cancer cells, suggesting that the function of this lincRNAs is exerted in the cytoplasm. Our results provided strong evidence supporting a hypothesis for cytoplasmic regulation, in which the lincRNA-ENST00000515084 rs12325489C>T SNP may affect the expression of this lincRNA by modifying the binding site for the miRNA-370. Our phenotypic experiment also demonstrated that the lincRNA-ENST00000515084 rs12325489C>T genotypes may significantly affect lincRNA-ENST00000515084 expression. Furthermore, evidence from our *in vitro* and *in* *vivo* study revealed that dysregulated expression of lincRNA correlated with tumor development.

In the present study, our result of association between a genetic polymorphism in the exonic regions of a lincRNA and susceptibility to breast cancer was firstly obtained from multiple independent case-control analyses derived from eastern, southern, and northern Chinese populations. Genotyping of these samples was performed in 3 independent laboratories. The relatively large sample sizes used decreased the size of the ORs that can be detected statistically. Moreover, we have achieved a study power of over 90% (two-sided test, α = 0.05) in detecting an OR of 1.79 for the rs12325489CT+CC genotypes (occurring at a frequency of 85.4% amongst the controls), when compared with the rs12325489TT genotype. Notably, the association is biologically plausible and is consistent with the findings of our functional studies.

In conclusion, the present study provided the first evidence that genetic polymorphisms in the exonic regions of lincRNAs play a role in mediating individual susceptibility to breast cancer. Our results further support the hypothesis that genetic variants in lincRNA exonic regions may alter microRNA-mediated regulation and that they are linked to an increased risk of breast cancer. Our findings warrant validation in larger, preferably population-based, case-control studies, as well as by well-designed mechanistic studies.

## Supporting Information

Figure S1 **Relative luciferase activity of the psiCHECK-2-rs12325489T and psiCHECK-2-rs12325489C constructs co-transfected with microRNAs (miRNA-1229, miRNA-1260b, miRNA-617, miRNA-1260) and inhibitor in breast cancer cells.** Renilla luciferase activity was measured and normalized to firefly luciferase. Six replicates were carried out for each group, and the experiment was repeated at least three times. Data are mean±standard error of the mean.
(TIF)

Figure S2 **miRNA-370 was constitutively expressed in breast cancer tissues harboring 3 different rs12325489C>T genotypes, respectively; data are mean±standard error of the mean, normalized to** ***U6*** **,** ***P*** **= 0.810.**
(TIF)

Table S1 **Summary of eligible studies considered in the study.**
(DOC)

Table S2 **The sequence of the predicted miRNA binding sites on the lincRNA sequence.**
(DOC)
