# Supplementary figures and images for: A Polymorphism rs12325489C>T in the LincRNA-ENST00000515084 Exon Was Found to Modulate Breast Cancer Risk via GWAS-Based Association Analyses
Source: PLoS One. 2014 May 30;9(5):e98251. doi: 10.1371/journal.pone.0098251 (PMC4039483; doi:10.1371/journal.pone.0098251)

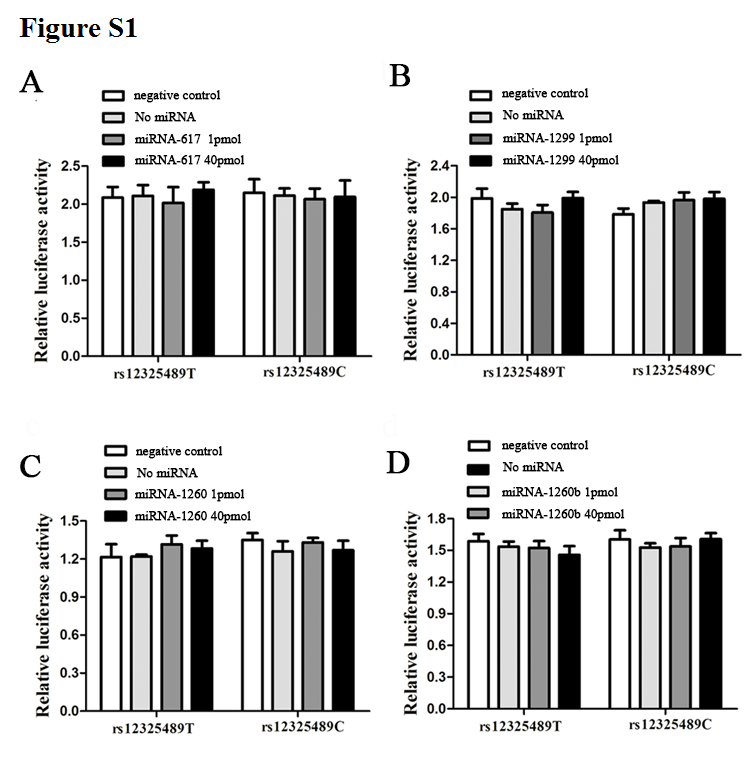

Supplement: Figure S1 — Relative luciferase activity of the psiCHECK-2-rs12325489T and psiCHECK-2-rs12325489C constructs co-transfected with microRNAs (miRNA-1229, miRNA-1260b, miRNA-617, miRNA-1260) and inhibitor in breast cancer cells. Renilla luciferase activity was measured and normalized to firefly luciferase. Six replicates were carried out for each group, and the experiment was repeated at least three times. Data are mean±standard error of the mean. (TIF) [file pone.0098251.s001.tif]

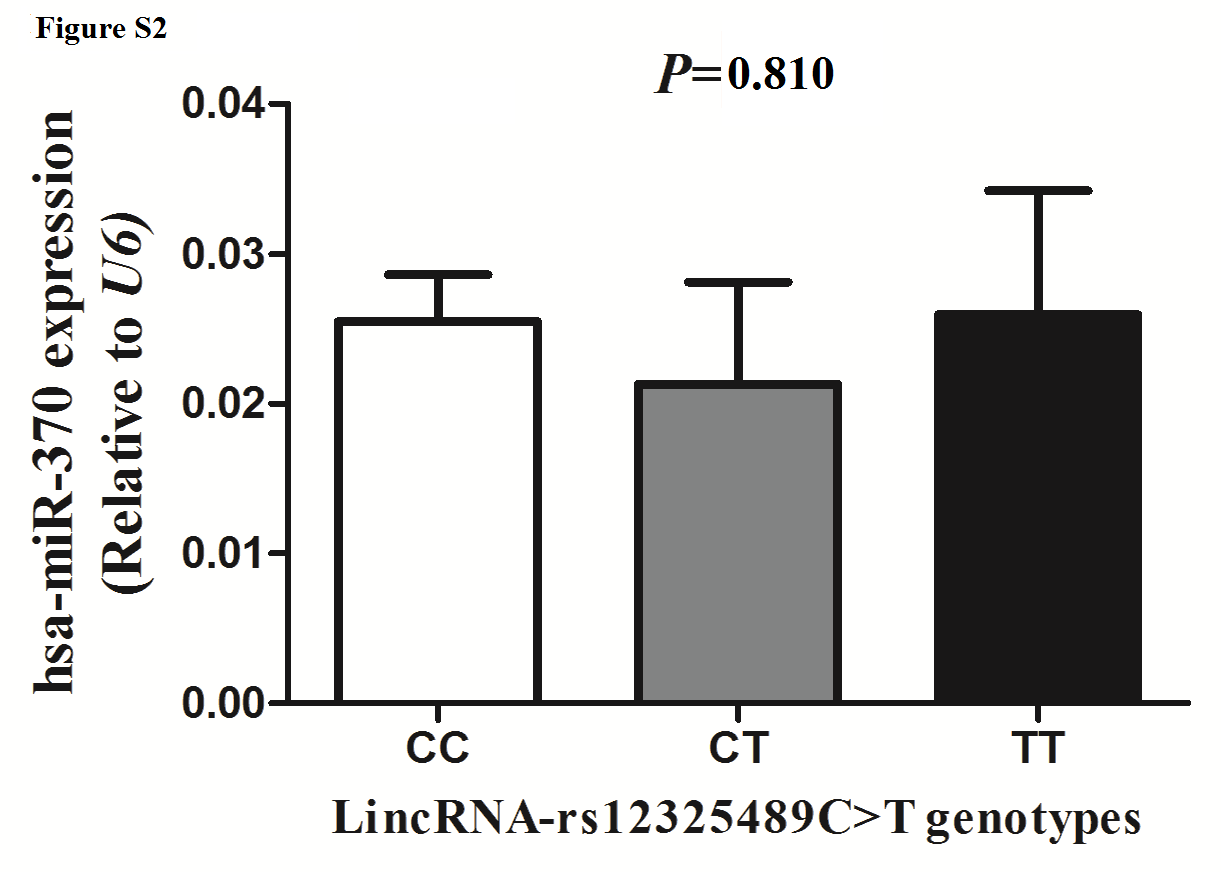

Supplement: Figure S2 — miRNA-370 was constitutively expressed in breast cancer tissues harboring 3 different rs12325489C>T genotypes, respectively; data are mean±standard error of the mean, normalized to U6 , P = 0.810. (TIF) [file pone.0098251.s002.tif]
