# Supplementary material for: A Polymorphism rs12325489C>T in the LincRNA-ENST00000515084 Exon Was Found to Modulate Breast Cancer Risk via GWAS-Based Association Analyses
Source: PLoS One. 2014 May 30;9(5):e98251. doi: 10.1371/journal.pone.0098251 (PMC4039483; doi:10.1371/journal.pone.0098251)
Supplement: Table S1 — Summary of eligible studies considered in the study. (DOC) [file pone.0098251.s003.doc]

| **Table S1. Summary of eligible studies considered in the study** | | | | | | | | | | | |
| --- | --- | --- | --- | --- | --- | --- | --- | --- | --- | --- | --- |
| **First author** | | **Year** | |  | **Ethnicity** |  | **Country** |  | **Chromosome** |  | **region^a^** |
| Long J |  | | 2012 |  | East-Asian |  | Chinese, Korean, and Japanese |  | 6q25.1 |  | 149.100.001-152.600.000 |
|  |  | |  |  |  |  |  |  | 11q24.3 |  | 127.400.001-130.300.000 |
| Cai Q |  | | 2011 |  | East-Asian |  | Chinese, Korean, and Japanese |  | 10q21.2 |  | 61.200.001-64.800.000 |
| Long J |  | | 2010 |  | Asian |  | Chinese, Japanese |  | 16q12.1 |  | 45.500.001-51.200.000 |
| Zheng W |  | | 2009 |  | Asian |  | Chinese |  | 6q25.1 |  | 149.100.001-152.600.000 |

**a From NCBI genome build 36**
