# Supplementary material for: A Polymorphism rs12325489C>T in the LincRNA-ENST00000515084 Exon Was Found to Modulate Breast Cancer Risk via GWAS-Based Association Analyses
Source: PLoS One. 2014 May 30;9(5):e98251. doi: 10.1371/journal.pone.0098251 (PMC4039483; doi:10.1371/journal.pone.0098251)
Supplement: Table S2 — The sequence of the predicted miRNA binding sites on the lincRNA sequence. (DOC) [file pone.0098251.s004.doc]

| **Sequence of the predicted miRNA binding sites** | **microRNA** | **Sequence of microRNA** | **Free energy(kcal/mol)** |
| --- | --- | --- | --- |
| acctggactttccatcaacatttgaagCggga | miRNA-370 | gccugcugggguggaaccuggu | -22.7 |
| tcaacatttgaagTgggagcagtcttgtggga | miRNA-1229 | cucucaccacugcccucccacag | -23.0 |
| tcaacatttgaagTgggagcagtcttgtggga | miRNA-1260b | aucccaccacugccaccau | -22.6 |
| ctttccatcaacatttgaagTgggagcagtct | miRNA-617 | agacuucccauuugaagguggc | -21.6 |
| tcaacatttgaagTgggagcagtcttgtggga | miRNA-1260 | aucccaccucugccacca | -20.5 |

**Table S2.** The sequence of the predicted miRNA binding sites on the lincRNA sequence
